# Mitochondrial dysfunction and apoptotic signaling induced by the combined action of 2-aminoethyl dihydrogen phosphate and methyl-β-cyclodextrin in melanoma cells

**DOI:** 10.3389/fphar.2025.1753894

**Published:** 2026-01-07

**Authors:** Thalles Anthony Duarte de Oliveira, Ranya Sthephanie Nascimento Ribeiro, Rosa Andrea Nogueira Laiso, Monique Gonçalves Alves, Yasmim Emilly Moreira Sousa, Ícaro Gabriel Teles Pacheco de Matos, Daniel da Conceição Rabelo, Rose Eli Grassi Rici, Sergio Mestieri Chammas, Solange Castro Afeche, Gustavo Henrique Doná Rodrigues Almeida, Durvanei Augusto Maria

**Affiliations:** 1 Graduate Program in Anatomy of Domestic and Wild Animals, School of Veterinary Medicine and Animal Science, University of São Paulo, São Paulo, Brazil; 2 Laboratory of Development and Innovation, Butantan Institute, São Paulo, Brazil; 3 Graduate Program in Medical Sciences, School of Medicine, University of São Paulo, São Paulo, Brazil; 4 Laboratory of Pharmacology, Butantan Institute, São Paulo, Brazil

**Keywords:** 2-AEH2P, apoptosis, bioenergetic stress, melanoma, metabolic vulnerability

## Abstract

Melanoma cells exhibit remarkable metabolic adaptability, sustained by lipid enrichment and mitochondrial resilience that enable survival under stress. Disrupting these bioenergetic and structural supports may represent an effective therapeutic avenue. This study investigated the antiproliferative, pro-apoptotic, and mitochondrial effects of 2-aminoethyl dihydrogen phosphate (2-AEH_2_P), alone and in combination with methyl-β-cyclodextrin (MβCD) in human (SK-MEL-28) and murine (B16-F10) melanoma cells, compared with normal human (FN1) and murine (L929) fibroblasts. Cell viability, proliferation index, mitochondrial membrane potential (ΔΨm), cell-cycle distribution, and apoptotic marker expression were evaluated following single and combined treatments. Morphological alterations were examined microscopically, and pharmacodynamic interaction was analyzed through drug-synergy assessment. 2-AEH_2_P displayed selective cytotoxicity toward melanoma cells, with markedly lower IC_50_ values than fibroblasts. Its combination with MβCD potentiated these effects, producing strong additive cytotoxicity. Treated melanoma cells showed distinct morphological alterations, including cytoplasmic projections and abnormal division, while fibroblasts preserved normal morphology. Combined treatments disrupted the cell-cycle profile, reducing G_0_/G_1_ and increasing S and G_2_/M phases, and induced mitochondrial dysfunction, evidenced by a significant decrease in ΔΨm. Expression of apoptotic markers (caspases-3 and -8, cytochrome c, p53, and Bad) increased, whereas anti-apoptotic Bcl-2 was downregulated. The combined use of 2-AEH_2_P and MβCD induced selective cytotoxicity in melanoma cells by disturbing lipid–mitochondrial homeostasis and activating intrinsic apoptotic signaling. These findings support a dual-target metabolic–membrane approach that exploits metabolic and mitochondrial vulnerabilities of melanoma and warrant further studies to elucidate its mechanisms and translational potential.

## 1 Introduction

Cutaneous melanoma remains one of the most aggressive forms of skin cancer, marked by rapid progression, pronounced heterogeneity, and poor responsiveness to conventional therapies (Leonardi et al., 2018; Waseh and Lee, 2023). In recent years, the incidence of melanoma has continued to rise globally, with increasing prevalence in younger adults and in regions with high ultraviolet exposure (Feng et al., 2025). Globally, more than 325,000 new cases and over 57,000 deaths were reported in 2024, and projections estimate that its burden will surpass 500,000 cases per year by 2040 if current trends persist (Arnold et al., 2022; Liu et al., 2024). Alarmingly, the disease increasingly affects younger adults, particularly in areas with high ultraviolet radiation exposure and fair-skinned populations, contributing to substantial public-health and economic impact (Arnold et al., 2022; Liu et al., 2024). Despite substantial advances in targeted therapy and immunomodulatory agents, long-term survival remains limited for many patients, particularly those who develop acquired resistance or progress under treatment. This therapeutic plateau is largely driven by melanoma’s capacity for metabolic rewiring, immune evasion, and microenvironmental remodeling, which allow tumor cells to bypass pathway inhibition and sustain proliferation despite pharmacological pressure (Ruocco et al., 2019). Resistance to BRAF/MEK inhibitors frequently arises through MAPK pathway reactivation, alternative RTK signaling, or mitochondrial metabolic compensation, whereas immune checkpoint resistance involves loss of antigen presentation, T-cell exclusion, or establishment of immunosuppressive niches. These adaptive mechanisms underscore the need for therapeutic approaches that extend beyond classical oncogene addiction and instead exploit conserved vulnerabilities, such as membrane organization, lipid homeostasis, and mitochondrial dependency (Kakadia et al., 2018; Kun et al., 2021). Beyond its genetic complexity, melanoma displays remarkable metabolic plasticity, enabling tumor cells to adapt and thrive under stress conditions that would normally impair survival. This adaptability is largely sustained by metabolic reprogramming and the capacity to remodel bioenergetic pathways and the tumor microenvironment (TME) to their advantage (Hsu et al., 2025; Shen et al., 2025). This metabolic adaptability represents an exploitable vulnerability that remains insufficiently targeted by current pharmacological approaches.

Melanoma depends on profound metabolic rewiring to sustain proliferation, resist oxidative stress, and evade apoptosis (Pizzimenti et al., 2021). Among its distinctive metabolic traits, melanoma exhibits abnormal reliance on lipid and cholesterol turnover. Tumor cells accumulate lipids to sustain membrane fluidity, enable lipid raft formation, and activate pro-survival cascades such as PI3K/Akt signaling (Pellerin et al., 2020; Chu et al., 2025). These cholesterol-rich nanodomains function as signaling platforms that orchestrate receptor clustering, oxidative balance, and apoptotic resistance (Singh et al., 2024; Zhang et al., 2024). Disrupting raft organization weakens oncogenic signaling, reduces metastatic competence, and sensitizes cells to apoptosis, suggesting that the lipid compartment of the plasma membrane represents a promising therapeutic axis.

In this context, methyl-β-cyclodextrin (MβCD) stands out as a pharmacological tool capable of depleting membrane cholesterol and altering the organization of lipid rafts, which can compromise membrane-associated signaling and induce apoptosis in highly proliferative cells (Păduraru et al., 2022; Ohno et al., 2023). Cholesterol extraction by MβCD disrupts the spatial organization of receptors and kinases housed within these microdomains, which may suppress pro-survival pathways, hinder cell migration, and promote apoptosis in highly proliferative cells. Additionally, cholesterol depletion can affect mitochondrial lipid composition, thereby further amplifying mitochondrial susceptibility to stress (Goicoechea et al., 2023). These mechanisms suggest that membrane remodeling is not merely a structural event but a metabolic perturbation capable of influencing intrinsic apoptotic signaling.

Complementing this membrane-targeted disruption, 2-aminoethyl dihydrogen phosphate (2-AEH_2_P), a monophosphate ester derived from ethanolamine, has been described as a modulator of phospholipid metabolism and mitochondrial integrity (Conceição et al., 2021; Alves et al., 2024). As a key intermediate of glycerophospholipid biosynthesis, 2-AEH_2_P interferes with pathways essential for maintaining membrane composition, organelle homeostasis, and bioenergetic balance. Previous studies have shown that 2-AEH_2_P promotes mitochondrial dysfunction, loss of membrane potential (ΔΨm), increased oxidative stress, and activation of intrinsic apoptosis (de Sousa Cabral et al., 2022). These effects are accompanied by altered expression of proteins involved in apoptotic commitment, such as cytochrome c, caspases, and the Bcl-2 family. Notably, 2-AEH_2_P demonstrates selectivity for tumor cells, which tend to rely more intensely on phospholipid turnover and mitochondrial flexibility to sustain rapid proliferation (de Sousa Cabral et al., 2022).

When associated with MβCD, 2-AEH_2_P may enhance the disruption of lipid–mitochondrial crosstalk, deepening bioenergetic stress and driving selective death in melanoma cells while preserving normal fibroblast viability (Giannitti et al., 2025). This dual-target approach, simultaneously destabilizing membrane architecture and compromising mitochondrial function, aligns with current trends in cancer pharmacology aimed at exploiting fundamental metabolic dependencies rather than individual oncogenic mutations. Such strategies may offer advantages in heterogeneous tumors like melanoma, where metabolic vulnerabilities are shared across genetically diverse subpopulations (Giannitti et al., 2025).

Based on that, this study aimed to evaluate the cytotoxic and apoptotic effects of the combined treatment with 2-AEH_2_P and MβCD in human (SK-MEL-28) and murine (B16-F10) melanoma cells. We hypothesized that this combination could exploit metabolic and mitochondrial vulnerabilities of melanoma, offering a multi-targeted pharmacological strategy that transcends classical cytotoxicity and addresses the energetic and structural dependencies that sustain tumor survival.

## 2 Materials and methods

**FIGURE 1:**
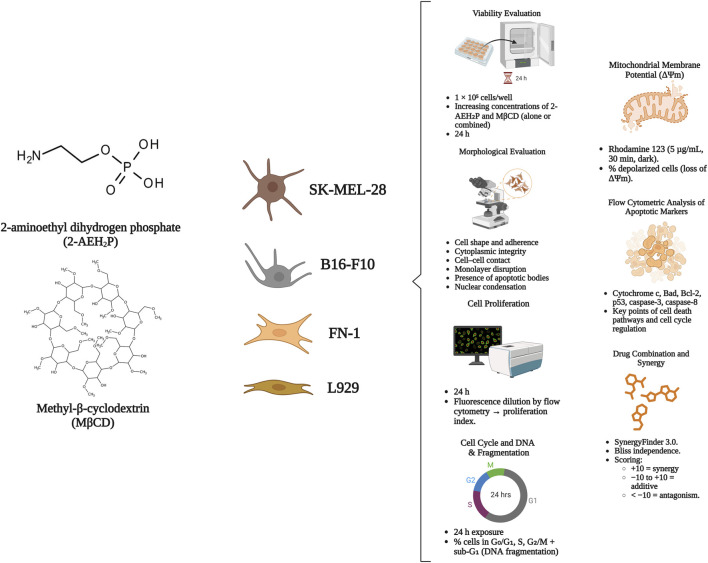
Workflow summarizing treatments, cell models, and mechanistic analyses. Melanoma cells (SK-MEL-28, B16-F10) and normal fibroblasts (FN-1, L929) were exposed for 24 h to increasing concentrations of 2-AEH_2_P and MBCD. After treatment, cytotoxicity, morphology, proliferation, cell-cycle progression, DNA fragmentation, and mitochondrial membrane potential (ΔΨm) were evaluated. Apoptotic markers were quantified by flow cytometry, and drug interactions were assessed using the Bliss independence model.

This study investigated the combined effects of 2-aminoethyl dihydrogen phosphate (2-AEH_2_P) with methyl-β-cyclodextrin (MβCD) on human and murine melanoma models. A series of biochemical and flow-cytometric assays were employed to assess cytotoxic potential, proliferative activity, alterations in cell-cycle dynamics, and apoptosis-related responses. Non-tumor fibroblasts were included as reference controls to determine selectivity. All assays were carried out in triplicate under standardized culture conditions. An overview of the experimental workflow is provided in Figure 1.

### 2.1 Preparation of 2-aminoethyl dihydrogen phosphate and methyl-β-cyclodextrin and cell culture conditions

The 2-aminoethyl dihydrogen phosphate (2-AEH_2_P) used in this study was obtained from PhosphoPure® (São Paulo, Brazil; Cat. No. PP-2AEH2P-100), supplied at certified purity (>99%) and accompanied by manufacturer’s analytical documentation. The compound, synthesized via controlled esterification, was dissolved in sterile ultrapure water to prepare a 100 mM stock solution, which was aliquoted and stored at 4 °C until use. Methyl-β-cyclodextrin (MβCD) was purchased from Sigma-Aldrich (St. Louis, MO, United States; Cat. No. C4555) and freshly dissolved in sterile phosphate-buffered saline (PBS, pH 7.4) to obtain a 50 mM stock solution, vortexed until complete dissolution, and filtered through a 0.22 µm sterile membrane before use. Human melanoma (SK-MEL-28; ATCC® HTB-72™) and murine melanoma (B16-F10; ATCC® RL-6475™) cell lines were employed as tumor models, whereas human fibroblasts (FN1; FMUSP 921/06) and murine fibroblasts (L929; ATCC® CCL-1™) were used as non-tumorigenic controls. FN1 and L929 fibroblasts were included as non-tumoral controls because they retain proliferative capacity, active membrane turnover, and metabolic plasticity, providing a physiologically relevant comparison to determine whether treatment effects reflect tumor-specific vulnerability rather than non-specific cytotoxicity (Peltier et al., 2022; Wieder, 2023; Duarte de Oliveira et al., 2025). Cells were maintained in RPMI-1640 medium (Thermo Fisher, Cat. No. 11875-093) supplemented with 2 mM L-glutamine (Gibco, Cat. No. 25030-081) 10 mM HEPES (Gibco, Cat. No. 15630-080), 24 mM sodium bicarbonate (Sigma-Aldrich, Cat. No. S5761), 1% antibiotic solution (100 U/mL penicillin and 100 μg/mL streptomycin), and 10% fetal bovine serum (Gibco, Cat. No. 16000-044). Cultures were incubated at 37 °C in a humidified atmosphere of 5% CO_2_, and cell viability was routinely verified using the Trypan Blue exclusion assay.

### 2.2 Cell viability assay

Cells were seeded in 6-well plates at a density of 1 × 10^5^ cells per well and allowed to adhere for 24 h under standard culture conditions. After attachment, cells were exposed to increasing concentrations of 2-aminoethyl dihydrogen phosphate (2-AEH_2_P; 1–100 mM) and methyl-β-cyclodextrin (MβCD; 0.1–5 mM) either individually or in combination. Cell viability was assessed after 24 h using the MTT [3-(4,5-dimethylthiazol-2-yl)-2,5-diphenyltetrazolium bromide] assay (Sigma-Aldrich, Cat. No. M5655). The MTT reagent was freshly prepared in phosphate-buffered saline (PBS, pH 7.4) at a final concentration of 5 mg/mL. Following treatment, the culture medium was removed and replaced with the MTT solution, followed by incubation for 3 h at 37 °C in a humidified atmosphere of 5% CO_2_. After incubation, formazan crystals formed by metabolically active cells were solubilized in methanol (Sigma-Aldrich, Cat. No. 34860), and absorbance was recorded at 570 nm using a microplate spectrophotometer. The half-maximal inhibitory concentration (IC_50_) values were obtained by non-linear regression analysis of dose–response curves generated in GraphPad Prism 7.0 (GraphPad Software, San Diego, CA, United States). The selectivity index (SI) was determined as the ratio between the IC_50_ value in fibroblasts and that in melanoma cells (SI = IC_50__fibroblast/IC_50__melanoma), representing the relative cytotoxic preference of the treatment for tumor cells over non-tumor controls. For single-agent assays, 2-AEH_2_P was evaluated in concentrations ranging from 1 to 100 mM and MβCD from 0.1 to 5 mM, based on preliminary dose–response curves and literature data. For combination studies, concentrations were adjusted according to the IC_50_ values individually determined for each compound in melanoma cells. The agents were co-administered at variable molar ratios to construct a complete dose–response matrix for synergy evaluation using the Bliss independence model.

### 2.3 Cell morphology analysis and selectivity index evaluation

Melanoma cells (SK-MEL-28 and B16-F10) and fibroblasts normal cells (FN1 and L929) were seeded in 24-well plates and incubated for 24 h to allow cell attachment. Following this period, cultures were exposed for an additional 24 h to increasing concentrations of 2-aminoethyl dihydrogen phosphate (2-AEH_2_P; 1–100 mM) and methyl-β-cyclodextrin (MβCD; 0.1–5 mM), either alone or in combination. After treatment, morphological alterations were examined using an inverted light microscope (Nikon Eclipse TS100, Tokyo, Japan) and documented photographically. Changes in cell shape, detachment, cytoplasmic granularity, membrane integrity, and apoptotic features were compared with untreated control cultures to qualitatively assess cytotoxic effects. The Selectivity Index (SI) was determined to evaluate the differential cytotoxicity of the compounds toward tumor and non-tumor cells, according to the equation:

$$\text{Selectivity}\;\text{Index}={\text{IC}}_{50\;\text{fibroblasts}}\;/\;{\text{IC}}_{50\;\text{melanoma}}$$

Where IC_50_ represents the concentration required to reduce cell viability by 50% relative to untreated controls. Higher SI values indicate increased selectivity toward tumor cells. For single-compound assays, 2-AEH_2_P and MβCD were tested individually at the concentrations described above. For combination assays, MβCD was applied at their half-maximal inhibitory concentrations (IC_50_), as well as at −25% and +50% of these values, in combination with graded concentrations of 2-AEH_2_P, allowing the evaluation of additive or synergistic interactions across a biologically relevant range. An SI value greater than 2 was interpreted as indicative of preferential cytotoxicity toward melanoma cells, in accordance with previously established criteria (Rashidi et al., 2017).

### 2.4 Cell cycle and DNA fragmentation analysis

Melanoma cells (SK-MEL-28 and B16-F10) and fibroblasts (FN1 and L929) were seeded in 24-well plates at a density of 1 × 10^5^ cells per well and incubated for 24 h to allow complete adhesion. After this period, cultures were exposed for an additional 24 h to different concentrations of 2-aminoethyl dihydrogen phosphate (2-AEH_2_P; 1–100 mM; Sigma-Aldrich, Cat. No. A5753) and methyl-β-cyclodextrin (MβCD; 0.1–5 mM; Sigma-Aldrich, Cat. No. C4555), either individually or in combination. Following treatment, cells were detached by trypsinization using 0.25% trypsin-EDTA (Gibco, Cat. No. 25200-056), washed twice with phosphate-buffered saline (PBS, pH 7.4; Gibco, Cat. No. 70013-016), and fixed in 70% cold ethanol containing RNase A (100 μg/mL; Sigma-Aldrich, Cat. No. R6513, DNase-free grade). Samples were stored at −20 °C until analysis. Before acquisition, cells were washed and resuspended in staining buffer containing 0.1% Triton X-100 (Sigma-Aldrich, Cat. No. T8787) and propidium iodide (PI, 50 μg/mL; Sigma-Aldrich, Cat. No. P4864) and incubated for 30 min in the dark at room temperature. DNA content was quantified on a FACSCanto II flow cytometer (BD Biosciences, San Jose, CA, United States), and cell-cycle phase distribution (G_0_/G_1_, S and G_2_/M) was determined using ModFit LT 6.0 software (Verity Software House, Topsham, ME, United States). For comparative assessment of treatment modulation, each cell line (human SK-MEL-28 and murine L929) was also treated with 50% of its respective IC_50_ concentration of 2-AEH_2_P, alone or in combination with MβCD, under identical conditions. A minimum of 10,000 gated events per sample were acquired and analyzed using CellQuest software (BD Biosciences), ensuring statistical robustness and reproducible phase resolution.

### 2.5 Assessment of cell proliferation using fluorescent dye labeling

The proliferative activity of melanoma cells (SK-MEL-28 and B16-F10) and fibroblasts (FN1 and L929) was evaluated using the fluorescent cell proliferation tracer carboxyfluorescein diacetate succinimidyl ester (CFSE-DA; Thermo Fisher Scientific, Cat. No. C34554). After 24 h of incubation to allow cell adherence and recovery, cultures were treated with increasing concentrations of 2-aminoethyl dihydrogen phosphate (2-AEH_2_P; 1–100 mM) and methyl-β-cyclodextrin (MβCD; 0.1–5 mM), either individually or in combination, at a seeding density of 2 × 10^5^ cells per well in 24-well plates. After 24 h of exposure, cells were detached using trypsin–EDTA solution (0.2% and 0.002%), centrifuged at 300 *g* for 5 min, and resuspended in fixation buffer (PBS containing 1% paraformaldehyde). Fluorescence intensity, inversely proportional to cell division rate, was measured by flow cytometry using a FACSCanto II system (BD Biosciences, San Jose, CA, United States). Data acquisition and proliferation profile quantification were performed using ModFit LT 6.0 software (Verity Software House, Topsham, ME, United States), enabling determination of the proliferation index and division rate for each treatment condition.

### 2.6 Evaluation of mitochondrial membrane potential (ΔΨm)

Melanoma cells (SK-MEL-28 and B16-F10) and fibroblasts (FN1 and L929) were seeded in 6-well plates at a density of 1 × 10^5^ cells per well and treated for 24 h with increasing concentrations of the experimental agents, either individually or in combination. After treatment, cells were collected by centrifugation at 300 *g* for 5 min and washed twice with phosphate-buffered saline (PBS, pH 7.4). Pellets were resuspended in FACS buffer (PBS supplemented with 1% bovine serum albumin). To evaluate mitochondrial membrane potential (ΔΨm), cells were incubated with rhodamine 123 (5 μg/mL; Molecular Probes/Thermo Fisher Scientific, Cat. R8004) for 30 min at 37 °C in the dark. After staining, excess dye was removed by washing twice with cold PBS, and samples were immediately analyzed on a FACSCalibur flow cytometer (BD Biosciences, San Jose, CA, United States) using 488 nm excitation and 530 ± 15 nm emission (FL1 channel). A minimum of 10,000 gated events per sample were acquired and analyzed using FlowJo software (version 10.7; BD Biosciences). Results were expressed as rhodamine 123 mean fluorescence intensity (MFI) relative to untreated control cells, where decreased MFI was interpreted as mitochondrial depolarization consistent with early mitochondrial dysfunction.

### 2.7 Flow cytometric analysis of cellular marker expression

Melanoma cells (SK-MEL-28 and B16-F10) and fibroblasts (FN1 and L929) were treated for 24 h with the experimental agents. After treatment, cells were harvested, washed twice with cold PBS (pH 7.4), fixed in 1% paraformaldehyde for 15 min at 4 °C, and permeabilized with 0.1% Triton X-100 in PBS for 10 min to enable intracellular antigen detection. Primary monoclonal antibodies targeting cytochrome c (clone 6H2.B4, BD Biosciences, Cat. 556432), Bad (Abcam, Cat. ab32445), Bcl-2 (clone 100/D5, BD Biosciences, Cat. 610538), p53 (clone DO-1, Santa Cruz Biotechnology, Cat. sc-126), caspase-3 (active form, BD Biosciences, Cat. 559565) and caspase-8 (clone 1C12, Cell Signaling Technology, Cat. 9,746) were applied at 1:50 for 1 h at 4 °C with gentle agitation. After washing in PBS with 0.2% BSA, detection was performed using species-appropriate fluorochrome-conjugated secondary antibodies (anti-rabbit IgG-FITC; anti-mouse IgG-PE; anti-goat IgG-APC), incubated for 30 min at 4 °C in the dark. All secondary antibodies used were polyclonal and validated for flow-cytometric applications, ensuring appropriate sensitivity and reproducibility for intracellular detection. A minimum of 10,000 gated events per sample were acquired on a FACSCanto II cytometer (BD Biosciences), and fluorescence intensities were analyzed using FCS Express 7.0 (*De Novo* Software). For combination assays, fixed sub-cytotoxic fractions of each agent’s IC_50_ were applied (≈12–12.5 mM 2-AEH_2_P plus 2–3 mM MβCD), enabling interrogation of their cooperative effects on membrane and mitochondrial disruption pathways. For mechanistic assays, each combination was administered at 50% of the respective IC_50_ values previously determined for each cell line. Accordingly, the concentrations used were 12.5 mM 2-AEH_2_P and 2.15 mM MβCD for SK-MEL-28, 12.2 mM 2-AEH_2_P and 2.95 mM MβCD for B16-F10, and 31.8 mM 2-AEH_2_P combined with 3.35 mM MβCD for FN1 and L929 fibroblasts. Quantification of protein modulation was derived from changes in mean fluorescence intensity (MFI) within viable, singlet populations, normalized to untreated controls, rather than absolute positivity thresholds, providing improved sensitivity for expression shift detection. Flow cytometry gating followed a standard approach including debris exclusion (FSC/SSC), singlet discrimination (FSC-H vs. FSC-A) and viable-cell gating before fluorescence quantification.

### 2.8 Synergy and drug interaction analysis

To investigate the interaction between 2-aminoethyl dihydrogen phosphate (2-AEH_2_P) and the compound methyl-β-cyclodextrin (MβCD), cells were exposed for 24 h to a series of independently varied concentrations. MβCD was applied at three levels: 25% below their respective IC_50_ values, at the IC_50_, and 50% above the IC_50_, combined with a concentration gradient of 2-AEH_2_P ranging from 1 to 100 mM. This experimental layout generated a multidimensional dose–response matrix that enabled a systematic examination of combined effects on cell viability. Quantitative interaction analyses were performed using the SynergyFinder 3.0 platform, which contrasts the experimentally observed inhibition rates with theoretical additive responses predicted by computational models. The Bliss independence model was selected to estimate the deviation between observed and expected outcomes, based on the premise that both agents act through mechanistically independent pathways. Synergy scores were interpreted according to established thresholds: values above +10 were considered indicative of synergism, between −10 and +10 as additive, and below −10 as antagonistic interactions. This method allowed a precise evaluation of whether co-administration of 2-AEH_2_P with MβCD potentiated, maintained, or attenuated cytotoxic activity relative to the single-compound treatments.

### 2.9 Statistical analyses

All data were expressed as mean ± standard deviation (SD) from three independent experiments performed in triplicate. Statistical analyses were conducted using GraphPad Prism version 7.0 (GraphPad Software, San Diego, CA, United States). Differences among experimental groups were analyzed using one-way ANOVA, followed by Tukey’s multiple comparison post-hoc test, which corrects for pairwise testing. Normality and variance assumptions were verified prior to applying ANOVA. A p-value ≤0.05 was considered statistically significant.

## 3 Results

### 3.1 Evaluation of the cytotoxic effects of 2-AEH_2_P and methyl-β-cyclodextrin (MβCD) in SK-MEL-28 and B16-F10 melanoma cells and FN1 and L929 fibroblasts

**FIGURE 2:**
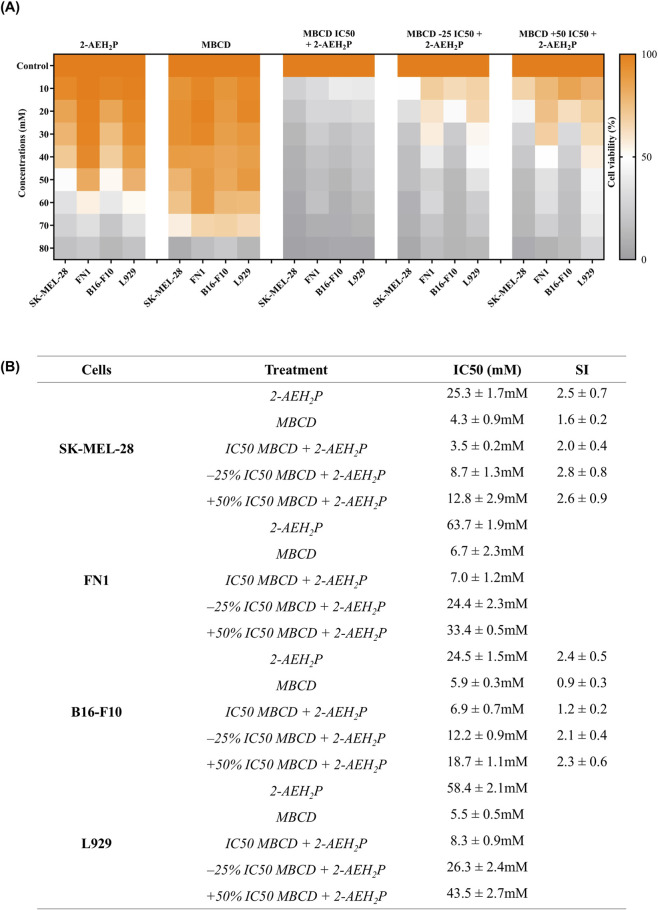
Cytotoxic effects and selectivity of 2-AEH2P and MβCD. Heatmap showing the viability of SK-MEL-28, B16-F10, FN-1, and L929 cells after 24 h of exposure to increasing concentrations of 2-AEH_2_P, MβCD, or their combined treatments **(A)**. The orange–white–gray scale represents relative viability values. Data represent mean values from three independent biological experiments, each performed in technical triplicate (n = 3). IC_50_ values (mM) and Selectivity Index (SI) for each treatment condition, comparing melanoma cells with non-tumor fibroblasts **(B)**. Values are expressed as mean ± standard deviation. Statistical comparisons were performed using one-way ANOVA followed by Tukey’s multiple-comparison post-hoc test, with p ≤ 0.05 considered statistically significant.

The monophosphate ester 2-AEH_2_P exhibited higher cytotoxicity toward melanoma cells than toward fibroblasts. In SK-MEL-28 cells, the IC_50_ was 25.3 ± 1.7 mM, compared with 63.7 ± 1.9 mM in FN1 fibroblasts. A similar pattern was observed in the murine model, with IC_50_ values of 24.5 ± 1.5 mM for B16-F10 melanoma cells and 58.4 ± 2.1 mM for L929 fibroblasts, confirming a consistent selectivity index for tumor cells (Figure 2). Treatment with isolated methyl-β-cyclodextrin (MβCD) also demonstrated greater cytotoxicity in melanoma cells. SK-MEL-28 cells showed an IC_50_ of 4.3 ± 0.9 mM, whereas FN1 fibroblasts exhibited a higher IC_50_ of 6.7 ± 2.3 mM. For B16-F10 cells, the IC_50_ was 5.9 ± 0.3 mM, while L929 fibroblasts showed 5.5 ± 0.5 mM, indicating sensitivity across both tumor and normal murine models.

The combined treatment with MβCD and 2-AEH_2_P enhanced cytotoxic activity in all melanoma models. In SK-MEL-28 cells, the combinations yielded IC_50_ values of 3.5 ± 0.2 mM, 8.7 ± 1.3 mM, and 12.8 ± 2.9 mM for the MβCD IC_50_ dose, −25%, and +50% concentration levels, respectively, when associated with 2-AEH_2_P. In FN1 fibroblasts, the corresponding IC_50_ values were 7.0 ± 1.2 mM, 24.4 ± 2.3 mM, and 33.4 ± 0.5 mM, demonstrating a markedly broader safety margin. For the B16-F10 model, combination IC_50_ values were 6.9 ± 0.7 mM, 12.2 ± 0.9 mM, and 18.7 ± 1.1 mM, while L929 fibroblasts displayed values of 8.3 ± 0.9 mM, 26.3 ± 2.4 mM, and 43.5 ± 2.7 mM under the same conditions. Although MβCD showed slightly lower IC_50_ values in melanoma compared to fibroblasts, this difference did not reach statistical significance, indicating that selective vulnerability becomes more evident in the combination context than under monotherapy.

To explicitly quantify potency and selectivity, we generated Table 1, which reports IC_50_ values with 95% confidence intervals and Selectivity Indexes for each treatment. These data confirm that 2-AEH_2_P and MβCD exhibit preferential cytotoxicity toward melanoma cells, particularly under combined regimens, consistent with the qualitative trends visualized in Figure 2.

**TABLE 1:** Half-maximal inhibitory concentration (IC_50_ ± SD), 95% confidence intervals, and Selectivity Index (SI) comparing melanoma (SK-MEL-28, B16-F10) and fibroblast (FN1, L929) responses to individual and combined treatments. Confidence intervals were obtained from non-linear regression analysis (t-distribution, n = 3).

Cell line	Treatment	IC_50_ (mM)	95% CI (mM)	SI
SK-MEL-28	2-AEH_2_P	25.3 ± 1.7	21.1 – 29.5	2.5 ± 0.7
	MβCD	4.3 ± 0.9	2.1 – 6.5	1.6 ± 0.2
	IC_50_ of MβCD (with fixed-dose 2-AEH_2_P)	3.5 ± 0.2	3.0 – 4.0	2.0 ± 0.4
	IC_50_ of MβCD (2-AEH_2_P at −25% of combination dose)	8.7 ± 1.3	5.5 – 11.9	2.8 ± 0.8
	IC_50_ of MβCD (2-AEH_2_P at +50% of combination dose)	12.8 ± 2.9	5.6 – 20.0	2.6 ± 0.9
FN1	2-AEH_2_P	63.7 ± 1.9	59.0 – 68.4	—
	MβCD	6.7 ± 2.3	1.0 – 12.4	—
	IC_50_ of MβCD (with fixed-dose 2-AEH_2_P)	7.0 ± 1.2	4.0 – 10.0	—
	IC_50_ of MβCD (2-AEH_2_P at −25% of combination dose)	24.4 ± 2.3	18.7 – 30.2	—
	IC_50_ of MβCD (2-AEH_2_P at +50% of combination dose)	33.4 ± 0.5	32.1 – 34.7	—
B16-F10	2-AEH_2_P	24.5 ± 1.5	20.8 – 28.2	2.4 ± 0.5
	MβCD	5.9 ± 0.3	5.2 – 6.6	0.9 ± 0.3
	IC_50_ of MβCD (with fixed-dose 2-AEH2P)	6.9 ± 0.7	5.2 – 8.6	1.2 ± 0.2
	IC_50_ of MβCD (2-AEH_2_P at −25% of combination dose)	12.2 ± 0.9	10.0 – 14.4	2.1 ± 0.4
	IC_50_ of MβCD (2-AEH_2_P at +50% of combination dose)	18.7 ± 1.1	15.9 – 21.5	2.3 ± 0.6
L929	2-AEH_2_P	58.4 ± 2.1	53.2 – 63.6	—
	MβCD	5.5 ± 0.5	4.3 – 6.8	—
	IC_50_ of MβCD (with fixed-dose 2-AEH_2_P)	8.3 ± 0.9	6.1 – 10.5	—
	IC_50_ of MβCD (2-AEH_2_P at −25% of combination dose)	26.3 ± 2.4	20.3 – 32.3	—
	IC_50_ of MβCD (2-AEH_2_P at +50% of combination dose)	43.5 ± 2.7	36.8 – 50.2	—

### 3.2 Morphological alterations in SK-MEL-28 and B16-F10 melanoma cells induced by 2-AEH_2_P and MβCD

After treatment with 2-AEH_2_P, SK-MEL-28 melanoma cells exhibited marked cytoplasmic projections, irregular cell borders, and abnormal division patterns, whereas FN1 fibroblasts preserved their typical elongated morphology. In B16-F10 melanoma cells, treatment resulted in irregular cytoplasmic extensions, loss of cell-to-cell contact, and a noticeable reduction in population density. In contrast, L929 fibroblasts showed no significant alterations in shape or density, indicating preserved structural integrity.

**FIGURE 3:**
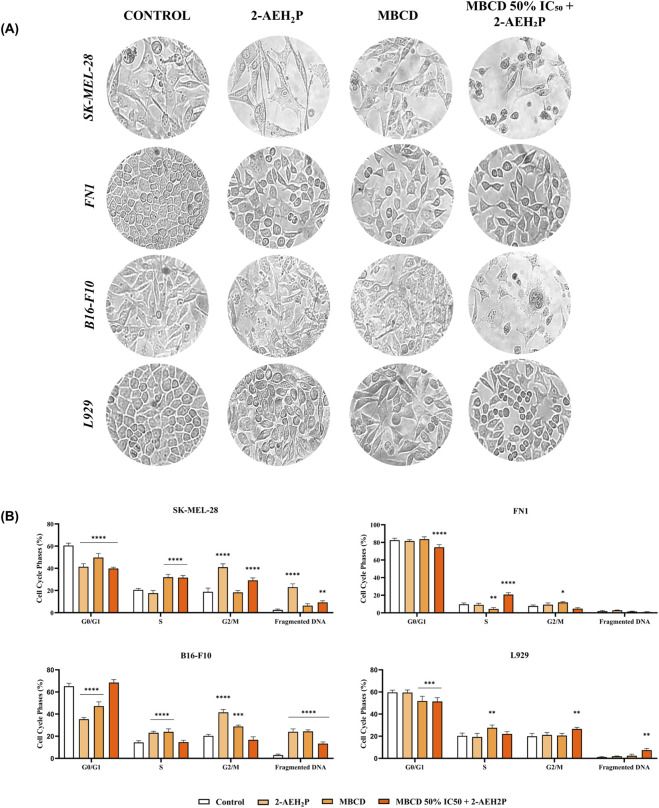
Morphological changes and cell-cycle alterations induced by 2-AEH2P and MβCD. Representative phase-contrast micrographs of SK-MEL-28 and B16-F10 melanoma cells and FN-1 and L929 fibroblasts after 24 h of treatment with 2-AEH_2_P, MβCD, or the combined protocol (MβCD 50% IC_50_ + 2-AEH_2_P). The images highlight alterations in adhesion, cytoplasmic organization, and nuclear morphology. Scale bars = 50 µm **(A)**. Cell-cycle distribution (G_0_/G_1_, S, G_2_/M, fragmented DNA) under the same experimental conditions **(B)**. Representative phase-contrast images of melanoma and fibroblast cultures (scale bar: 50 µm). Data are expressed as mean ± SD from three independent biological experiments, each performed in technical triplicate (n = 3). Statistical analysis was performed by one-way ANOVA followed by Tukey’s post-hoc test. Significance versus untreated control: *p < 0.05, **p < 0.01, ***p < 0.001, ****p < 0.0001.

Treatment with MβCD produced mild morphological changes in SK-MEL-28 cells when applied alone; however, more pronounced alterations were observed when MβCD was used at its IC_50_ concentration or in combination with 2-AEH_2_P. These conditions induced spherical and atypical cell contours, cytoplasmic retraction, and decreased adherence. FN1 fibroblasts exhibited reduced cell density after MβCD exposure but maintained normal morphological characteristics (Figure 3A).

In B16-F10 melanoma cells, MβCD also affected cytoskeletal organization, with more pronounced structural disruption at the IC_50_ concentration and in association with 2-AEH_2_P. The combined treatment intensified these effects in melanoma cells, characterized by rounded morphology, loss of polarity, and membrane irregularities, while fibroblasts continued to display preserved morphology despite modest reductions in confluence.

### 3.3 Cell-cycle phase modulation in melanoma and fibroblasts exposed to 2-AEH_2_P and MβCD

After isolated treatment with 2-AEH_2_P, both melanoma and fibroblast cells exhibited changes in cell-cycle phase distribution, although the magnitude of the effects differed markedly between the models. In SK-MEL-28 melanoma cells, 2-AEH_2_P reduced the proportion of cells in G_0_/G_1_ (−19.1%), accompanied by an increase in the S phase (+11.4%) and a substantial accumulation in G_2_/M (+22.2%). These shifts indicate disruption of normal cell-cycle progression and suggest arrest at the G_2_/M checkpoint. In contrast, FN1 fibroblasts showed only minimal modulation, with slight increases in G_0_/G_1_ (+0.9%) and G_2_/M (+1.5%) and a modest reduction in S phase (−0.6%). DNA fragmentation significantly increased in SK-MEL-28 cells (20.5%), whereas FN1 fibroblasts showed no meaningful change (1.1% ± 0.2%), reinforcing the selective pro-apoptotic effect of 2-AEH_2_P.

Isolated MβCD treatment also altered cell-cycle distribution in melanoma. In SK-MEL-28 cells, MβCD reduced the G_0_/G_1_ population (−10.9%) and increased the S phase (+11.4%), indicating interference with DNA synthesis and checkpoint control. In B16-F10 melanoma cells, G_0_/G_1_ decreased even more markedly (−18.0%) with a corresponding rise in S phase (+9.5%). FN1 fibroblasts, however, showed moderate increases in G_0_/G_1_ (+7.8%) and S phase (+7.2%), with only a slight change in G_2_/M (+0.6%), suggesting a cytostatic rather than cytotoxic response. DNA fragmentation increased in both melanoma lines (3.9% in SK-MEL-28% and 21.4% in B16-F10), whereas fibroblasts again showed no significant alterations (Figure 3B). These sub-G1 values correspond to hypodiploid apoptotic nuclei quantified after debris exclusion, rather than extracellular debris.

### 3.4 Effects of 2-AEH_2_P and MβCD on proliferation dynamics in melanoma and fibroblast models

**FIGURE 4:**
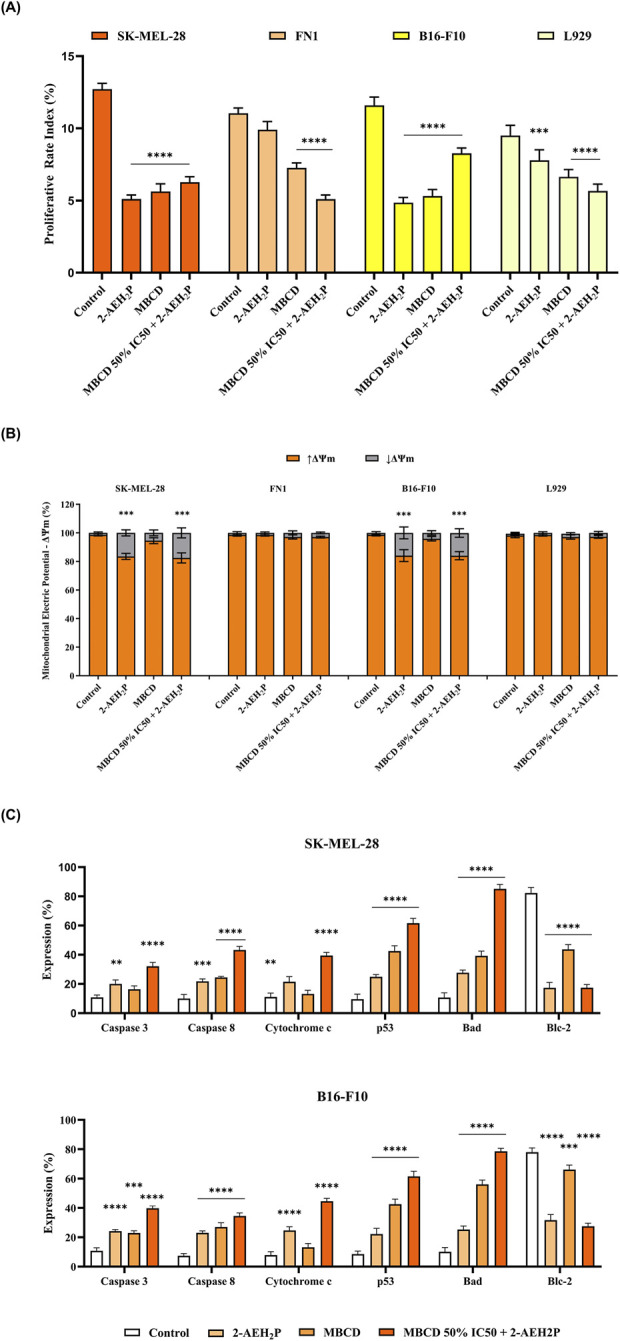
Effects of 2-AEH2P and MβCD on proliferation, mitochondrial function, and apoptotic markers. Proliferation analysis showing how SK-MEL-28, B16-F10, FN-1, and L929 cells respond after 24 h of exposure to 2-AEH_2_P, MβCD, or the combined regimen (MβCD 50% IC_50_ + 2-AEH_2_P). The proliferative rate index demonstrates distinct reductions depending on the treatment and cell type **(A)**. Evaluation of mitochondrial membrane potential (ΔΨm) indicating shifts in the proportion of cells with preserved or reduced polarization following treatment, revealing differential mitochondrial sensitivity across melanoma and fibroblast lines **(B)**. Flow-cytometric quantification of key apoptotic regulators, caspase-3, caspase-8, cytochrome c, p53, Bad, and Bcl-2, showing treatment-dependent modulation of pro- and anti-apoptotic pathways in SK-MEL-28 and B16-F10 cells **(C)**. Values represent relative changes in MFI compared to untreated control. Data are expressed as mean ± SD from three independent biological experiments, each conducted in technical triplicate (n = 3). Statistical analysis was performed using one-way ANOVA followed by Tukey’s multiple-comparison test. Significance versus control: *p < 0.05, **p < 0.01, ***p < 0.001, ****p < 0.0001.

Cell proliferation analysis (Figure 4A) showed that isolated treatment with 2-AEH_2_P markedly reduced the proliferation index (PI) of melanoma cells. SK-MEL-28 cells presented a PI of 4.9 ± 0.9 and B16-F10 cells a PI of 5.1 ± 1.5. In contrast, fibroblast viability was less affected, with FN1 and L929 cells showing higher PI values (9.5 ± 0.9 and 7.2 ± 1.5, respectively), indicating a selective antiproliferative effect. Isolated treatment with methyl-β-cyclodextrin (MβCD) also reduced proliferation, although to different extents across the models. SK-MEL-28 cells exhibited a PI of 5.2 ± 1.4, while B16-F10 melanoma cells showed a stronger response (PI = 5.6 ± 0.9). In fibroblasts, the effects remained modest, with PI values of 7.0 ± 0.8 for FN1 and 6.2 ± 1.3 for L929. The combined treatment with 2-AEH_2_P and MβCD further modulated cell proliferation. SK-MEL-28 cells showed a PI of 6.5 ± 1.1, whereas FN1 fibroblasts exhibited a PI of 4.9 ± 0.5. For the B16-F10 model, the combination resulted in a PI of 8.5 ± 1.0, while L929 fibroblasts showed a PI of 5.3 ± 1.2.

### 3.5 Mitochondrial membrane potential modulation in melanoma and fibroblasts exposed to 2-AEH_2_P and MβCD

Analysis of mitochondrial membrane potential (ΔΨm) (Figure 4B) showed that isolated treatment with 2-AEH_2_P induced a marked reduction in ΔΨm in both melanoma cell lines. SK-MEL-28 cells exhibited a decrease of 15.1% ± 0.5%, and B16-F10 cells showed a similar reduction of 14.6% ± 1.5%. In contrast, FN1 and L929 fibroblasts demonstrated minimal sensitivity, with reductions of only 0.2% ± 0.1% and 0.3% ± 0.2%, respectively, indicating preserved mitochondrial integrity in non-tumor cells. Isolated MβCD also promoted mitochondrial depolarization, although to a lesser extent. SK-MEL-28 and B16-F10 cells showed decreases of 4.1% ± 1.4% and 2.8% ± 0.3%, respectively. When MβCD was combined with 2-AEH_2_P, ΔΨm loss was strongly enhanced in both melanoma models, reaching 16.1% ± 0.4% in SK-MEL-28% and 14.7% ± 1.0% in B16-F10 cells. Fibroblasts again displayed minimal alterations, with FN1 showing reductions of 1.4% ± 0.2% (isolated) and 1.5% ± 0.1% (association), and L929 showing 1.1% ± 0.2% and 1.5% ± 0.2% under the same conditions.

### 3.6 Modulation of intrinsic apoptotic by 2-AEH_2_P and MβCD in melanoma cells

Treatment with 2-AEH_2_P activated key components of the intrinsic apoptotic pathway in both human and murine melanoma cells (Figure 4C). In SK-MEL-28 cells, 2-AEH_2_P increased the expression of caspase-3 (9.2% ± 1%) and caspase-8 (11.8% ± 0.5%), accompanied by elevated cytochrome c release (10.4% ± 1.4%). Upstream regulators were also modulated, with p53 expression rising by 15.3% ± 0.4% and Bad increasing by 17% ± 0.6%. Conversely, the anti-apoptotic protein Bcl-2 was markedly reduced (−64.9% ± 1.9%). A similar profile was observed in B16-F10 cells, with increased caspase-3 (10.8% ± 0.3%), caspase-8 (7.4% ± 0.4%), cytochrome c (7.8% ± 0.9%), p53 (8.5% ± 1.9%), and Bad (10.2% ± 1.1%), together with a pronounced reduction in Bcl-2 (−78% ± 1.8%).

Isolated MβCD treatment induced moderate activation of apoptotic markers in SK-MEL-28 cells, whereas its association with 2-AEH_2_P markedly amplified these responses. Caspase-3 increased from 5.6% ± 0.8% (isolated) to 21.3% ± 1.1% (association), and caspase-8 showed a similar potentiation (14.5% ± 0.3% vs. 33.3% ± 1.2%). Cytochrome c release rose sharply under the combined treatment (28.3% ± 0.9%), compared to a minimal effect with MβCD alone (2% ± 1.2%). p53 expression increased by 33% ± 1.4% with isolated MβCD and by 52.1% ± 1.6% with the combined treatment. Bad expression also rose significantly under the association (74.4% ± 1.8%) compared to isolated MβCD (28.6% ± 1.7%). Consistent with these pro-apoptotic changes, Bcl-2 expression decreased more strongly in the combined treatment (−64.8% ± 0.9%) than in the isolated condition (−38.6% ± 1.5%). In B16-F10 cells, the combined treatment likewise produced greater activation of the apoptotic cascade than either agent alone. Caspase-3 expression increased from 12.2% ± 0.3% (isolated) to 30% ± 0.5% (association), and caspase-8 rose from 19.7% ± 1.1% to 27.2% ± 0.8%. Cytochrome c release was markedly elevated in the combined treatment (36.8% ± 1.2%) compared to the isolated condition (5.3% ± 0.9%). p53 expression increased from 34% ± 1.4% to 53% ± 1.6% under the combined treatment, while Bad expression rose from 45.9% ± 1% to 68.4% ± 1.3%. In line with these pro-apoptotic shifts, Bcl-2 expression was reduced by −11.8% ± 1.5% in the isolated treatment and by −50.5% ± 1.1% in the association.

### 3.7 Evaluation of pharmacological interaction between 2-AEH_2_P and MβCD in melanoma cells

**FIGURE 5:**
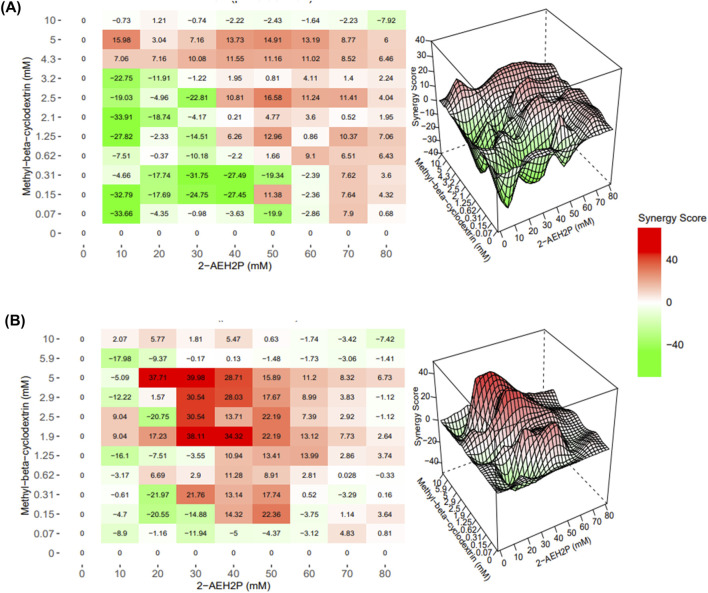
Interaction profile of 2-AEH2P and MβCD in melanoma cells. Bliss independence–based interaction maps for SK-MEL-28 cells illustrating how different dose combinations of 2-AEH_2_P and MβCD modulate cytotoxic responses across the concentration matrix **(A)**. The corresponding visualization for B16-F10 cells **(B)** displays interaction distribution patterns, highlighting zones of additive enhancement and localized response intensification across the evaluated dose ranges rather than widespread synergy. These surfaces aid in interpreting how complementary perturbation of membrane organization and phospholipid metabolism contributes to measurable increases in melanoma sensitivity under combined exposure.

Synergy analyses (Figure 5) revealed that the combined treatment with MβCD and 2-AEH_2_P produced consistent additive interactions in both melanoma cell lines. In SK-MEL-28 cells, the combination yielded an average synergy score of 7.83 ± 1.2, indicating predominantly additive effects across the concentration range. A comparable pattern was observed in B16-F10 melanoma cells, with an average synergy score of 7.58 ± 0.4, likewise consistent with an additive interaction profile. In fibroblast models, the combination also demonstrated an overall additive pattern. FN1 fibroblasts showed mild inhibitory responses at higher concentrations, resulting in an average synergy score of −2.87 ± 1.3, still within the additive range. L929 fibroblasts exhibited small regions of interaction classified by the software as synergistic, although the overall average score remained −3.31 ± 1.7, again corresponding to an additive effect. Thus, despite clear potentiation, the interaction pattern remained strongly additive rather than synergistic.

## 4 Discussion

The monophosphate ester 2-AEH_2_P has demonstrated high efficacy in inducing selective cytotoxicity in melanoma cells, as confirmed by the present findings. This compound primarily interferes with phospholipid metabolism and membrane turnover rather than directly targeting DNA, which makes it a promising alternative for tumors resistant to conventional genotoxic agents (Ferreira et al., 2011; Senft and Ronai, 2016; Alves et al., 2021). Previous studies have demonstrated its property to induce apoptosis in multiple tumor models as human breast adenocarcinoma MCF-7, and chronic myeloid leukemia K562 (Conceição et al., 2021; Alves et al., 2021; de Sousa Cabral et al., 2022; Alves et al., 2024; Duarte de Oliveira et al., 2025), reinforcing that the compound operates through a conserved antitumoral mechanism. Our results demonstrated that 2-AEH_2_P exhibits preferential cytotoxicity toward melanoma cells, producing substantially higher effects in SK-MEL-28 and B16-F10 cells than in non-tumoral fibroblasts. This selective toxicity, supported by a favorable therapeutic index, indicates that tumor cells are considerably more sensitive to alterations in membrane dynamics and phospholipid-dependent signaling than normal fibroblasts (Tan et al., 2017). The compound also induced G2/M arrest in melanoma cells, a finding consistent with prior evidence showing that 2-AEH_2_P promotes cell cycle blockade and increases DNA fragmentation in highly proliferative tumor lines (Ferreira et al., 2013). Since melanoma cells rely on rapid membrane synthesis and remodeling to sustain proliferation, interfering with these pathways may be particularly effective to limit tumor expansion.

Mitochondrial involvement was also evident, as treatment with 2-AEH_2_P caused a significant reduction in mitochondrial membrane potential in melanoma cells, while no significant alterations were observed in fibroblasts. This finding implies that melanoma cells, especially drug-resistant variants, depend more on mitochondrial oxidative metabolism than on glycolysis, making mitochondrial destabilization a critical vulnerability (Du et al., 2025). The dissipation of ΔΨm observed in this study was associated with cytochrome c release, upregulation of p53 and Bad, and suppression of Bcl-2, reinforcing the activation of the intrinsic apoptotic pathway as a central mechanism triggered by membrane and metabolic destabilization (Wu and Bratton, 2013; Westaby et al., 2021; Mustafa et al., 2024).

While the study demonstrates mitochondrial depolarization and apoptotic commitment following combined treatment, the molecular link between membrane perturbation and mitochondrial collapse remains to be fully defined. Given that cholesterol-rich domains regulate signaling platforms and mitochondrial contact sites, it is plausible that MβCD-driven raft destabilization amplifies mitochondrial stress induced by 2-AEH_2_P, ultimately contributing to ΔΨm loss (Ziolkowski et al., 2010; Mollinedo and Gajate, 2021). Future work incorporating reactive oxygen species (ROS) profiling, redox sensor assays, or lipidomics will help determine whether oxidative imbalance or alterations in membrane phospholipid composition serve as upstream mediators of the mitochondrial dysfunction observed here. Such analyses may refine the mechanistic model and reveal additional vulnerabilities exploitable for melanoma targeting.

Methyl-β-cyclodextrin (MβCD), which promotes cholesterol depletion and disrupts lipid raft integrity, also demonstrated cytotoxicity in melanoma cells, with more intense effects in SK-MEL-28 (Mohammad et al., 2014). These observations are in line with evidence that cholesterol removal increases membrane fluidity and compromises survival signaling, particularly in tumors that depend on raft-associated pathways such as PI3K/Akt (Manuela et al., 2019; Bai et al., 2021). When combined with 2-AEH_2_P, MβCD intensified cytotoxic effects, reduced the proliferation index, increased G2/M accumulation, and markedly amplified mitochondrial depolarization in melanoma cells (Duarte de Oliveira et al., 2025). However, Bliss interaction analysis indicated that this enhancement reflected a predominantly additive rather than synergistic profile. This pattern likely arises from parallel but not fully cooperative mechanisms, whereby phospholipid metabolism disruption by 2-AEH_2_P and raft destabilization by MβCD converge on mitochondrial stress without amplifying each other sufficiently to generate supra-additive synergy (Mitchell et al., 2022). The combined response is best interpreted as strong additive potentiation driven by complementary disruption of lipid–mitochondrial homeostasis.

The combination treatment also increased caspase-3, caspase-8, cytochrome c, p53, and Bad expression, while significantly reducing Bcl-2, indicating that dual targeting of membrane structure and mitochondrial integrity triggers a stronger apoptotic response than either treatment alone (Vogler et al., 2025). Importantly, fibroblasts remained largely unaffected by the combined treatment, which is consistent with their lower dependence on cholesterol-rich domains and slower membrane remodeling kinetics (Barillé-Nion et al., 2020; Makimoto et al., 2021; Zlotnikov et al., 2023). The data support a mechanistic model in which 2-AEH_2_P destabilizes phospholipid synthesis and membrane turnover, MβCD disrupts cholesterol-dependent membrane microdomains, and the combined perturbation propagates inwardly to the mitochondria, culminating in the activation of intrinsic apoptosis (Ulloth et al., 2007; Szydlarska et al., 2018). This dual membrane–mitochondria targeting strategy appears to selectively compromise melanoma cells while sparing normal fibroblasts, suggesting translational potential for combinatorial therapeutic approaches (Awad and Abdul Karim, 2025). This mechanistic interpretation aligns with emerging evidence that melanoma relies disproportionately on oxidative metabolism, lipid biogenesis, and mitochondrial adaptability to sustain survival and therapeutic resistance (Tan et al., 2024). Unlike many malignancies that maintain a glycolytic bias, melanoma frequently shifts toward oxidative phosphorylation under stress, implying that mitochondrial destabilization represents a clinically relevant vulnerability (Kim et al., 2025). In this context, 2-AEH_2_P is particularly attractive because it perturbs phospholipid biosynthesis and mitochondrial membrane stability, whereas MβCD disrupts cholesterol-rich raft platforms that serve as signaling hubs and regulate mitochondrial–endoplasmic reticulum contact sites (Conceição et al., 2021; Alves et al., 2021; de Sousa Cabral et al., 2022; Duarte de Oliveira et al., 2025). This combination amplifies apoptotic stress and undermines melanoma viability in a biologically meaningful manner. While our findings indicate that membrane cholesterol depletion and phospholipid metabolism interference converge functionally at mitochondrial destabilization, this mechanistic intersection remains inferential; resolving the precise biochemical linkage will require lipidomic profiling and signaling interrogation beyond the scope of the present proof-of-concept study.

Although the findings provide clear mechanistic insights, certain limitations must be acknowledged. One limitation is that the study evaluated only a single human melanoma cell line (SK-MEL-28) and one murine line (B16-F10) (Vincent and Postovit, 2017; Lopes et al., 2022). While both are widely used and biologically relevant, melanoma exhibits substantial genetic and metabolic heterogeneity, and the inclusion of additional human cell lines could further validate the generalizability of the observed effects (Vincent and Postovit, 2017). Nevertheless, using two evolutionarily distinct models strengthens the internal consistency of the findings without compromising their biological plausibility. Another limitation concerns the supraphysiological concentrations of 2-AEH_2_P required *in vitro* to elicit cytotoxic effects, which are unlikely to be achieved through systemic administration (de Sousa Cabral et al., 2022). It should also be acknowledged that the IC_50_ values observed are in the millimolar range, which represents a clear translational limitation and reflects the *in-vitro* conditions and physicochemical profile of membrane-active compounds. Likewise, our work focused on mechanistic outcomes *in vitro* and did not address downstream metabolic flux alterations or *in vivo* relevance. It should also be noted that the current findings were obtained in SK-MEL-28 and B16-F10 models, and therefore cannot be extrapolated to all melanoma backgrounds; validation in genetically diverse human melanoma lines, particularly BRAF-mutant models, will be required before broader claims of shared metabolic vulnerability can be made (Aktary et al., 2023; Michielon et al., 2023). Additionally, systemic administration of MβCD carries dose-limiting toxicity and uncertain membrane-targeting selectivity *in vivo*, indicating that pharmacological translation of our findings will require controlled delivery formats or locoregional approaches to mitigate off-target effects and enhance therapeutic index (Khatoon et al., 2025). These considerations indicate that the present findings should be interpreted primarily as mechanistic proof-of-concept, rather than as immediately translatable dosing regimens. Nevertheless, the study was designed to elucidate interactions between phospholipid metabolism and membrane integrity, and the robust additive effects observed across independent melanoma models provide a strong biological foundation for translational progress (Kook and Kim, 2024). Future investigations will explore strategies capable of overcoming pharmacokinetic barriers, including locoregional delivery, formulation-based enhancement, and preliminary melanoma xenograft models, to determine whether the selective cytotoxicity observed here can be reproduced under physiological exposure constraints (Liu et al., 2018). Importantly, none of these limitations undermine the central conclusion that dual targeting of membrane architecture and mitochondrial integrity selectively compromises melanoma viability. These results position dual lipid–metabolic interference as a rational direction for melanoma therapy development.

## 5 Conclusion

This study demonstrated that the combined use of 2-aminoethyl dihydrogen phosphate (2-AEH_2_P) and methyl-β-cyclodextrin (MβCD) produced strong additive cytotoxic effects against melanoma cells. MβCD potentiated the action of 2-AEH_2_P by perturbing cholesterol-rich membrane domains, affecting lipid raft stability and facilitating apoptotic signaling, particularly in SK-MEL-28 and B16-F10 cells. The combination also induced a marked reduction in mitochondrial membrane potential (ΔΨm), supporting mitochondrial dysfunction as a key downstream event in this response. While melanoma cells displayed pronounced morphological and biochemical changes, normal fibroblasts largely retained viability and structural integrity, reinforcing the selective nature of the interaction. These results suggest that dual perturbation of phospholipid metabolism and membrane organization constitutes a promising metabolic–membrane targeting approach for melanoma. Nonetheless, additional validation in genetically diverse human melanoma models will be important to further substantiate the translational applicability of these findings.

## Data Availability

The raw data supporting the conclusions of this article will be made available by the authors, without undue reservation.

## References

[B1] Aktary Z. Raymond J. H. Pouteaux M. Delmas V. Petit V. Larue L. (2023). Derivation and use of cell lines from mouse models of melanoma. J. Investigative Dermatology 143, 538–544.e2. 10.1016/J.JID.2023.01.005 36958885

[B2] Silva M. G. L. Cabral L. G. S. Alves M. G. Conceição T. O. Hesse H. Nogueira Laiso R. A. (2021). 2-aminoethyl dihydrogen phosphate as a modulator of proliferative and apoptotic effects in breast cancer cell lines. J. Pharm. Pharmacol. 9, 83–97.

[B3] Alves M. G. Cabral L. G. S. Totti P. G. F. Azarias F. R. Pomini K. T. Rici R. E. G. (2024). 2-Aminoethyl dihydrogen phosphate (2-AEH2P) associated with cell metabolism-modulating drugs presents a synergistic and pro-apoptotic effect in an *in vitro* model of the ascitic ehrlich tumor. Biomedicines 12, 109. 10.3390/BIOMEDICINES12010109 38255214 PMC10813795

[B4] Arnold M. Singh D. Laversanne M. Vignat J. Vaccarella S. Meheus F. (2022). Global burden of cutaneous melanoma in 2020 and projections to 2040. JAMA Dermatol 158, 495–503. 10.1001/JAMADERMATOL.2022.0160 35353115 PMC8968696

[B5] Awad A. M. A. M. Abdul Karim N. (2025). Dysregulation of mitochondrial function in cancer cells. Int. J. Mol. Sci. 26, 6750. 10.3390/IJMS26146750 40724998 PMC12295676

[B6] Bai H. Wang J. Phan C. U. Chen Q. Hu X. Shao G. (2021). Cyclodextrin-based host-guest complexes loaded with regorafenib for colorectal cancer treatment. Nat. Commun. 12 (12), 759. 10.1038/s41467-021-21071-0 33536421 PMC7858623

[B7] Barillé-Nion S. Lohard S. Juin P. P. (2020). Targeting of BCL-2 family members during anticancer treatment: a necessary compromise between individual cell and ecosystemic responses? Biomolecules 10, 1109. 10.3390/BIOM10081109 32722518 PMC7464802

[B8] Chu Z. Fang L. Xiang Y. Ding Y. (2025). Research progress on cholesterol metabolism and tumor therapy. Discov. Oncol. 2025 16 (16), 1–23. 10.1007/S12672-025-02430-5 40307614 PMC12043555

[B9] Conceição T. O. Cabral L. G. S. Laveli-Silva M. G. Pacheco J. C. Alves M. G. Rabelo D. C. (2021). New potential antiproliferative monophosphoester 2-aminoethyl dihydrogen phosphate in K-562 and K-562 MDR+ leukemia cells. Biomed. & Pharmacother. 142, 112054. 10.1016/J.BIOPHA.2021.112054 34463267

[B10] de Sousa Cabral L. G. Hesse H. Freire K. A. de Oliveira C. S. Pedron C. N. Alves M. G. (2022). The BR2 peptide associated with 2-aminoethyl dihydrogen phosphate is a formulation with antiproliferative potential for a triple-negative breast cancer model. Biomed. & Pharmacother. 153, 113398. 10.1016/J.BIOPHA.2022.113398 36076530

[B11] Du H. Xu T. Yu S. Wu S. Zhang J. (2025). Mitochondrial metabolism and cancer therapeutic innovation. Signal Transduct. Target. Ther. 10 (10), 245. 10.1038/s41392-025-02311-x 40754534 PMC12319113

[B12] Duarte de Oliveira T. A. Rodrigues Almeida G. H. D. Ribeiro R. S. N. Laiso R. A. N. Alves M. G. Sousa Y. E. M. (2025). Synergistic antiproliferative and pro-apoptotic activity of dacarbazine combined with 2-aminoethyl dihydrogen phosphate in melanoma cells. Biomed. Pharmacother. 193, 118866. 10.1016/J.BIOPHA.2025.118866 41337881

[B13] Feng F. Ma Y. Zhao Y. Wan Z. Zhang R. Yang S. (2025). Global assessment of surface ultraviolet radiation and malignant skin melanoma incidence from 1990 to 2021. Sci. Rep. 15 (15), 39300. 10.1038/s41598-025-23066-z 41214082 PMC12602703

[B14] Ferreira A. K. Meneguelo R. Neto S. C. Chierice G. O. Maria D. A. (2011). Synthetic phosphoethanolamine induces apoptosis through Caspase-3 pathway by decreasing expression of bax/bad protein and changes cell cycle in melanoma. J. Cancer Sci. Ther. 3, 1–7. 10.4172/1948-5956.1000058

[B15] Ferreira A. K. Meneguelo R. Pereira A. Filho O. M. R. Chierice G. O. Maria D. A. (2013). Synthetic phosphoethanolamine induces cell cycle arrest and apoptosis in human breast cancer MCF-7 cells through the mitochondrial pathway. Biomed. Pharmacother. 67, 481–487. 10.1016/j.biopha.2013.01.012 23773853

[B16] Giannitti G. Paganoni A. J. J. Marchesi S. Garavaglia R. Fontana F. (2025). Mitochondrial bioenergetics and networks in melanoma: an update. Apoptosis 30, 2042–2056. 10.1007/S10495-025-02155-4 40721981 PMC12474643

[B17] Goicoechea L. Conde de la Rosa L. Torres S. García-Ruiz C. Fernández-Checa J. C. (2023). Mitochondrial cholesterol: metabolism and impact on redox biology and disease. Redox Biol. 61, 102643. 10.1016/J.REDOX.2023.102643 36857930 PMC9989693

[B18] Hsu C. Y. Ahmed Y. K. mohammed S. Alghamdi M. A. AL-Ghamdi H. S. Mohammed J. S. (2025). Metabolism at the core of melanoma: from bioenergetics to immune escape and beyond. Semin. Oncol. 52, 152413. 10.1016/j.seminoncol.2025.152413 40945210

[B19] Kakadia S. Yarlagadda N. Awad R. Kundranda M. Niu J. Naraev B. (2018). Mechanisms of resistance to BRAF and MEK inhibitors and clinical update of US food and drug Administration-approved targeted therapy in advanced melanoma. Onco Targets Ther. 11, 7095–7107. 10.2147/OTT.S182721 30410366 PMC6200076

[B20] Khatoon H. Faudzi S. M. M. Sohajda T. (2025). Mechanisms and therapeutic applications of β-Cyclodextrin in drug solubilisation and delivery systems. Chem. Biodivers. 22, e00359. 10.1002/CBDV.202500359 40616835

[B21] Kim Y. Doma V. Çakır U. Kuras M. Betancourt L. H. Pla I. (2025). Mitochondrial proteome landscape unveils key insights into melanoma severity and treatment strategies. Cancer 131, e35897. 10.1002/CNCR.35897 40545870 PMC12183497

[B22] Kook E. Kim D. H. (2024). Elucidating the role of lipid-metabolism-related signal transduction and inhibitors in skin cancer. Metabolites 14, 309–314. 10.3390/METABO14060309 38921444 PMC11205519

[B23] Kun E. Tsang Y. T. M. Ng C. W. Gershenson D. M. Wong K. K. (2021). MEK inhibitor resistance mechanisms and recent developments in combination trials. Cancer Treat. Rev. 92, 102137. 10.1016/J.CTRV.2020.102137 33340965

[B24] Leonardi G. C. Falzone L. Salemi R. Zanghì A. Spandidos D. A. Mccubrey J. A. (2018). Cutaneous melanoma: from pathogenesis to therapy. Int. J. Oncol. 52, 1071–1080. (Review). 10.3892/IJO.2018.4287 29532857 PMC5843392

[B25] Liu Q. Das M. Liu Y. Huang L. (2018). Targeted drug delivery to melanoma. Adv. Drug Deliv. Rev. 127, 208–221. 10.1016/J.ADDR.2017.09.016 28939379

[B26] Liu C. Liu X. Hu L. Li X. Xin H. Zhu S. (2024). Global, regional, and national burden of cutaneous malignant melanoma from 1990 to 2021 and prediction to 2045. Front. Oncol. 14, 1512942. 10.3389/FONC.2024.1512942 39777336 PMC11703817

[B27] Lopes J. Rodrigues C. M. P. Gaspar M. M. Reis C. P. (2022). How to treat melanoma? The current status of innovative nanotechnological strategies and the role of minimally invasive approaches like PTT and PDT. Pharmaceutics 14, 1817. 10.3390/PHARMACEUTICS14091817 36145569 PMC9504126

[B28] Makimoto A. Fang J. Maeda H. (2021). Development of a selective tumor-targeted drug delivery system: hydroxypropyl-acrylamide polymer-conjugated pirarubicin (P-THP) for pediatric solid tumors. Cancers (Basel) 13, 3698. 10.3390/CANCERS13153698 34359599 PMC8345214

[B29] Manuela Garcia Laveli da Silva Knop L. B. Maria D. A. (2019). Meclizine chloridrate and Methyl-β-Cyclodextrin associated with monophosphoester synthetic phosphoethanolamine modulating proliferative potential in triple-negative breast cancer cells. J. Pharm. Pharmacol. 7. 10.17265/2328-2150/2019.07.006

[B30] Michielon E. López González M. Stolk D. A. Stolwijk J. G. C. Roffel S. Waaijman T. (2023). A reconstructed human melanoma-in-skin model to study immune modulatory and angiogenic mechanisms facilitating initial melanoma growth and invasion. Cancers (Basel) 15, 2849. 10.3390/CANCERS15102849/S1 37345186 PMC10216824

[B31] Mitchell W. Tamucci J. D. Ng E. L. Liu S. Birk A. V. Szeto H. H. (2022). Structure-activity relationships of mitochondria-targeted tetrapeptide pharmacological compounds. Elife 11, e75531. 10.7554/ELIFE.75531 35913044 PMC9342957

[B32] Mohammad N. Malvi P. Meena A. S. Singh S. V. Chaube B. Vannuruswamy G. (2014). Cholesterol depletion by methyl-β-cyclodextrin augments tamoxifen induced cell death by enhancing its uptake in melanoma. Mol. Cancer 13, 204. 10.1186/1476-4598-13-204 25178635 PMC4175626

[B33] Mollinedo F. Gajate C. (2021). Mitochondrial targeting involving cholesterol-rich lipid rafts in the mechanism of action of the antitumor ether lipid and alkylphospholipid analog edelfosine. Pharmaceutics 13, 763. 10.3390/PHARMACEUTICS13050763 34065546 PMC8161315

[B34] Mustafa M. Ahmad R. Tantry I. Q. Ahmad W. Siddiqui S. Alam M. (2024). Apoptosis: a comprehensive overview of signaling pathways, morphological changes, and physiological significance and therapeutic implications. Cells 13, 1838. 10.3390/CELLS13221838 39594587 PMC11592877

[B35] Ohno Y. Toshino M. Mohammed A. F. A. Fujiwara Y. Komohara Y. Onodera R. (2023). Mannose-methyl-β-cyclodextrin suppresses tumor growth by targeting both colon cancer cells and tumor-associated macrophages. Carbohydr. Polym. 305, 120551. 10.1016/j.carbpol.2023.120551 36737200

[B36] Păduraru D. N. Niculescu A. G. Bolocan A. Andronic O. Grumezescu A. M. Bîrlă R. (2022). An updated overview of cyclodextrin-based drug delivery systems for cancer therapy. Pharmaceutics 14, 1748. 10.3390/PHARMACEUTICS14081748 36015374 PMC9412332

[B37] Pellerin L. Carrié L. Dufau C. Nieto L. Ségui B. Levade T. (2020). Lipid metabolic reprogramming: Role in melanoma progression and therapeutic perspectives. Cancers (Basel) 12, 3147. 10.3390/CANCERS12113147 33121001 PMC7692067

[B38] Peltier A. Seban R. D. Buvat I. Bidard F. C. Mechta-Grigoriou F. (2022). Fibroblast heterogeneity in solid tumors: from single cell analysis to whole-body imaging. Semin. Cancer Biol. 86, 262–272. 10.1016/J.SEMCANCER.2022.04.008 35489628

[B39] Pizzimenti S. Ribero S. Cucci M. A. Grattarola M. Monge C. Dianzani C. (2021). Oxidative stress-related mechanisms in melanoma and in the acquired resistance to targeted therapies. Antioxidants 10. 10.3390/ANTIOX10121942 34943045 PMC8750393

[B40] Rashidi M. Seghatoleslam A. Namavari M. Amiri A. Fahmidehkar M. A. Ramezani A. (2017). Selective cytotoxicity and apoptosis-induction of Cyrtopodion scabrum extract against digestive cancer cell lines. Int. J. Cancer Manag. 10 (10), e8633. 10.5812/IJCM.8633

[B41] Ruocco M. R. Avagliano A. Granato G. Vigliar E. Masone S. Montagnani S. (2019). Metabolic flexibility in melanoma: a potential therapeutic target. Semin. Cancer Biol. 59, 187–207. 10.1016/J.SEMCANCER.2019.07.016 31362075

[B42] Senft D. Ronai Z. A. (2016). Regulators of mitochondrial dynamics in cancer. Curr. Opin. Cell Biol. 39, 43–52. 10.1016/J.CEB.2016.02.001 26896558 PMC4828329

[B43] Shen D. Zhang L. Li S. Tang L. (2025). Metabolic reprogramming in melanoma therapy. Cell Death Discov. 11, 308. 10.1038/S41420-025-02617-3 40617808 PMC12228840

[B44] Singh M. K. Han S. Kim S. Kang I. (2024). Targeting lipid metabolism in cancer stem cells for anticancer treatment. Int. J. Mol. Sci. 2024, 11185. 10.3390/IJMS252011185 39456967 PMC11508222

[B45] Szydlarska J. Weiss C. Marycz K. (2018). The effect of Methyl-β-cyclodextrin on apoptosis, proliferative activity, and oxidative stress in adipose-derived mesenchymal stromal cells of horses suffering from metabolic Syndrome (EMS). Molecules 2018, 287. 10.3390/MOLECULES23020287 29385746 PMC6017619

[B46] Tan L. T. H. Chan K. G. Pusparajah P. Lee W. L. Chuah L. H. Khan T. M. (2017). Targeting membrane lipid a potential cancer cure? Front. Pharmacol. 8, 12. 10.3389/FPHAR.2017.00012 28167913 PMC5253362

[B47] Tan I. J. Parikh A. K. Cohen B. A. (2024). Melanoma metabolism: molecular mechanisms and therapeutic implications in cutaneous oncology. Cancer Med. 13, e70386. 10.1002/CAM4.70386 39494561 PMC11532834

[B48] Ulloth J. E. Almaguel F. G. Padilla A. Bu L. Liu J. W. De Leon M. (2007). Characterization of methyl-β-cyclodextrin toxicity in ngf-differentiated pc12 cell death. Neurotoxicology 28, 613–621. 10.1016/J.NEURO.2007.01.001 17292476 PMC1994916

[B49] Vincent K. M. Postovit L. M. (2017). Investigating the utility of human melanoma cell lines as tumour models. Oncotarget 8, 10498–10509. 10.18632/ONCOTARGET.14443 28060736 PMC5354675

[B50] Vogler M. Braun Y. Smith V. M. Westhoff M. A. Pereira R. S. Pieper N. M. (2025). The BCL2 family: from apoptosis mechanisms to new advances in targeted therapy. Signal Transduct. Target. Ther. 10 (10), 91. 10.1038/s41392-025-02176-0 40113751 PMC11926181

[B51] Waseh S. Lee J. B. (2023). Advances in melanoma: epidemiology, diagnosis, and prognosis. Front. Med. (Lausanne) 10, 1268479. 10.3389/FMED.2023.1268479/FULL 38076247 PMC10703395

[B52] Westaby D. Jimenez-Vacas J. M. Padilha A. Varkaris A. Balk S. P. de Bono J. S. (2021). Targeting the intrinsic apoptosis pathway: a window of opportunity for prostate cancer. Cancers (Basel) 14, 51. 10.3390/CANCERS14010051 35008216 PMC8750516

[B53] Wieder R. (2023). Fibroblasts as turned agents in cancer progression. Cancers (Basel) 15, 2014. 10.3390/CANCERS15072014 37046676 PMC10093070

[B54] Wu C. C. Bratton S. B. (2013). Regulation of the intrinsic apoptosis pathway by reactive oxygen species. Antioxid. Redox Signal 19, 546–558. 10.1089/ARS.2012.4905 22978471 PMC3717204

[B55] Zhang H. Li Y. Huang J. Shen L. Xiong Y. (2024). Precise targeting of lipid metabolism in the era of immuno-oncology and the latest advances in nano-based drug delivery systems for cancer therapy. Acta Pharm. Sin. B 14, 4717–4737. 10.1016/J.APSB.2024.07.021 39664426 PMC11628863

[B56] Ziolkowski W. Szkatula M. Nurczyk A. Wakabayashi T. Kaczor J. J. Olek R. A. (2010). Methyl-beta-cyclodextrin induces mitochondrial cholesterol depletion and alters the mitochondrial structure and bioenergetics. FEBS Lett. 584, 4606–4610. 10.1016/J.FEBSLET.2010.10.023 20965172

[B57] Zlotnikov I. D. Dobryakova N. V. Ezhov A. A. Kudryashova E. V. (2023). Achievement of the selectivity of cytotoxic agents against cancer cells by creation of combined formulation with terpenoid adjuvants as prospects to overcome multidrug resistance. Int. J. Mol. Sci. 24, 8023. 10.3390/IJMS24098023/S1 37175727 PMC10178335

