# Supplementary material for: Mitochondrial dysfunction and apoptotic signaling induced by the combined action of 2-aminoethyl dihydrogen phosphate and methyl-β-cyclodextrin in melanoma cells
Source: Front Pharmacol. 2026 Jan 7;16:1753894. doi: 10.3389/fphar.2025.1753894 (PMC12819607; doi:10.3389/fphar.2025.1753894)
Supplement: Supplementary file 1 [file Supplementaryfile1.docx]

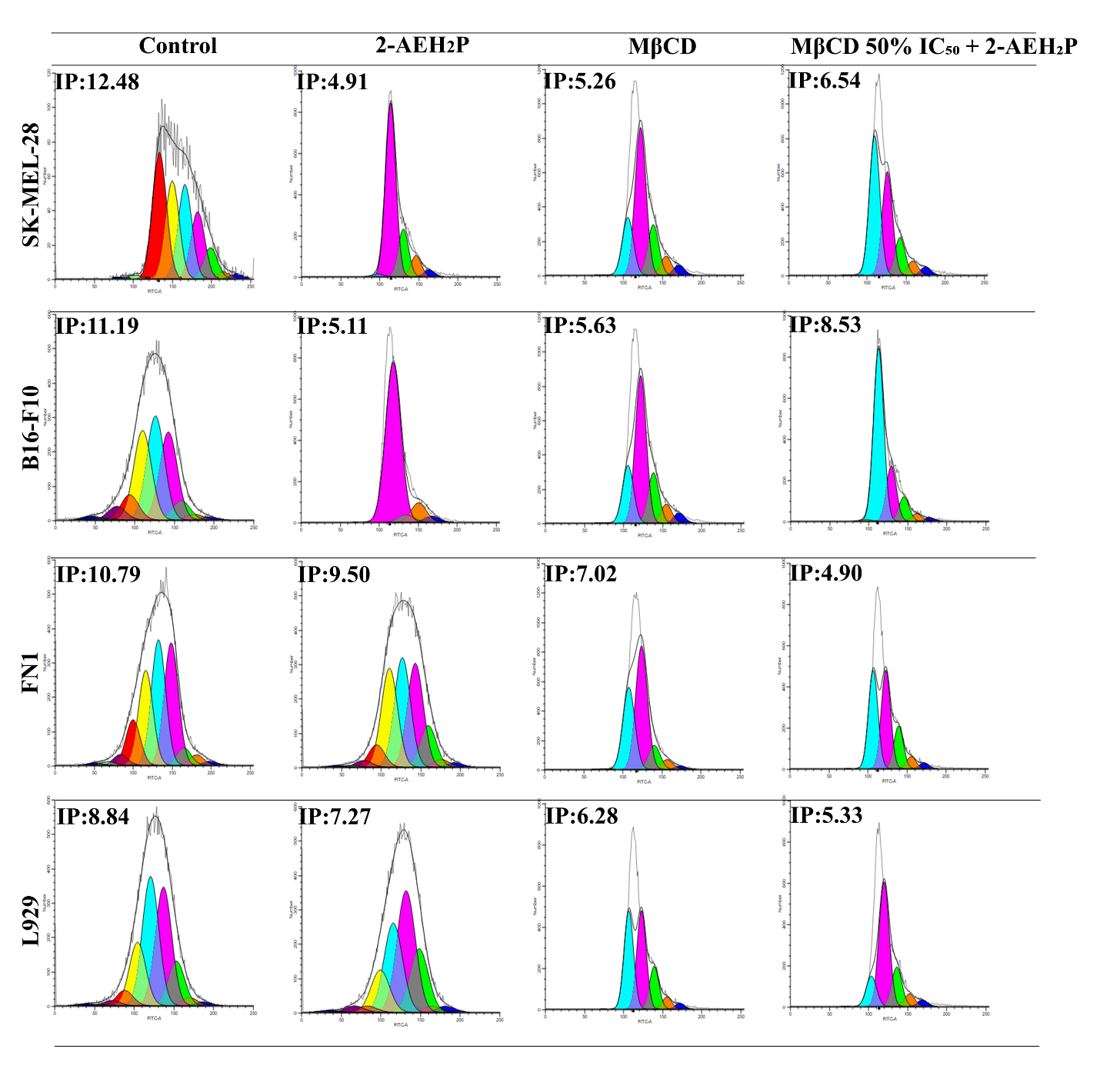


**Supplementary Figure 1.** Representative flow cytometry proliferation profiles of melanoma cells and fibroblasts following treatment with 2-AEH₂P, MβCD, and their combination. Representative CFSE-based proliferation histograms for SK-MEL-28, B16-F10, FN1, and L929 cells after 24 h of exposure to 2-AEH₂P, MβCD, or the combined regimen (MβCD at 50% IC₅₀ plus 2-AEH₂P). Control samples show the expected progressive dilution of CFSE fluorescence, whereas treated groups exhibit reduced dye dilution consistent with decreased proliferation. Each color peak represents a successive cell division cycle, and the proliferation index (PI) for each condition is shown above its respective plot. Data illustrate the selective antiproliferative effect of the treatments on melanoma cells compared with fibroblasts.


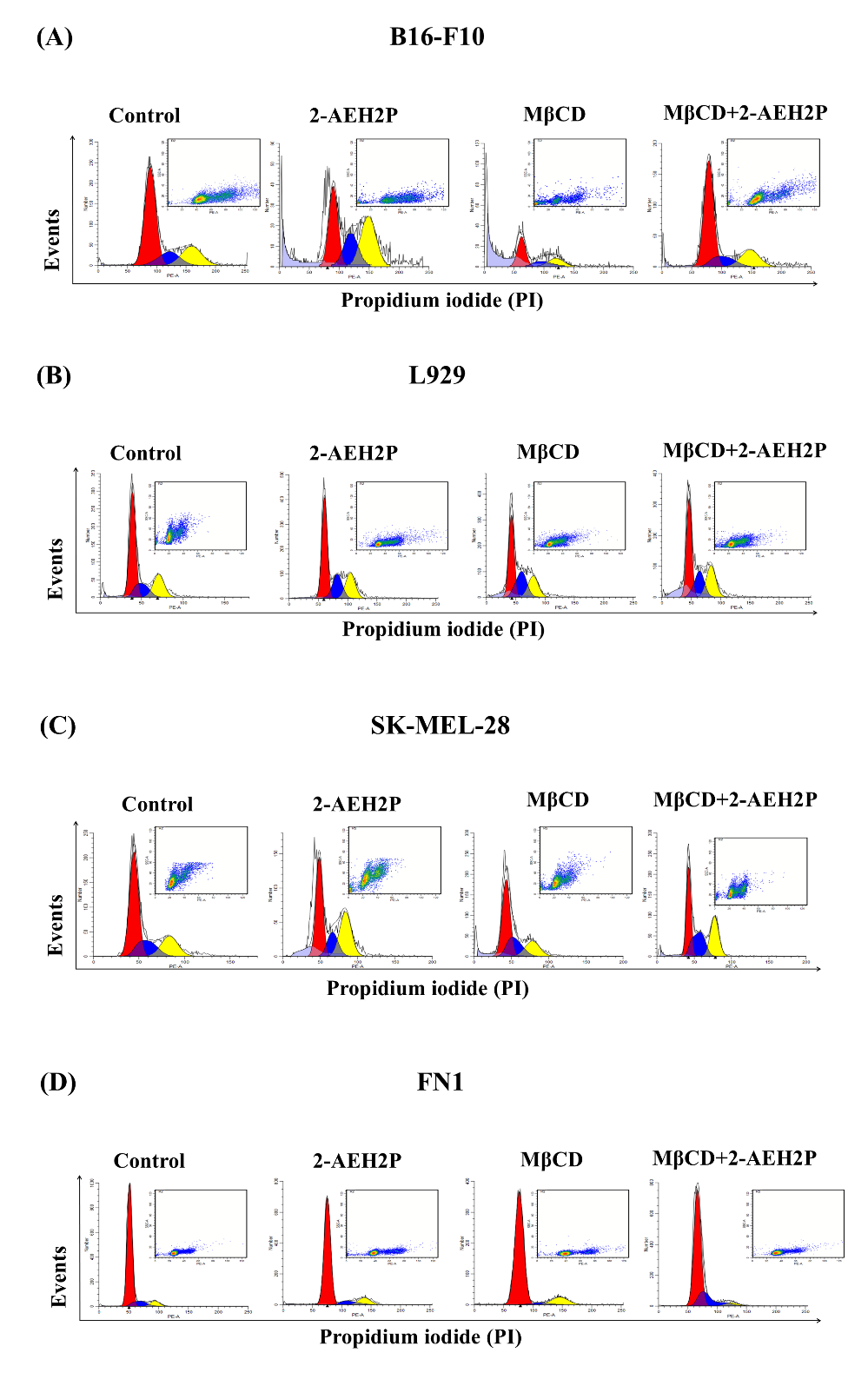


**Supplementary Figure 2.** Representative cell cycle histograms of melanoma cells and fibroblasts following treatment with 2-AEH₂P, MβCD, and their combination. (A) B16-F10, (B) L929, (C) SK-MEL-28, and (D) FN1 cells were stained with propidium iodide (PI) after 24 h of treatment with 2-AEH₂P, MβCD, or the combined regimen. Representative DNA content histograms display the distribution of cells across G0/G1, S, and G2/M phases, with sub-G1 peaks indicating apoptotic DNA fragmentation. Insets show forward/side scatter gating used to define viable singlet populations prior to PI quantification. Treatments produced distinct shifts in cell cycle profiles, with melanoma cells exhibiting increased sub-G1 fractions and reduced S-phase content, while fibroblasts showed comparatively modest alterations. These data illustrate differential sensitivity of tumor versus non-tumor cells to the agents evaluated.


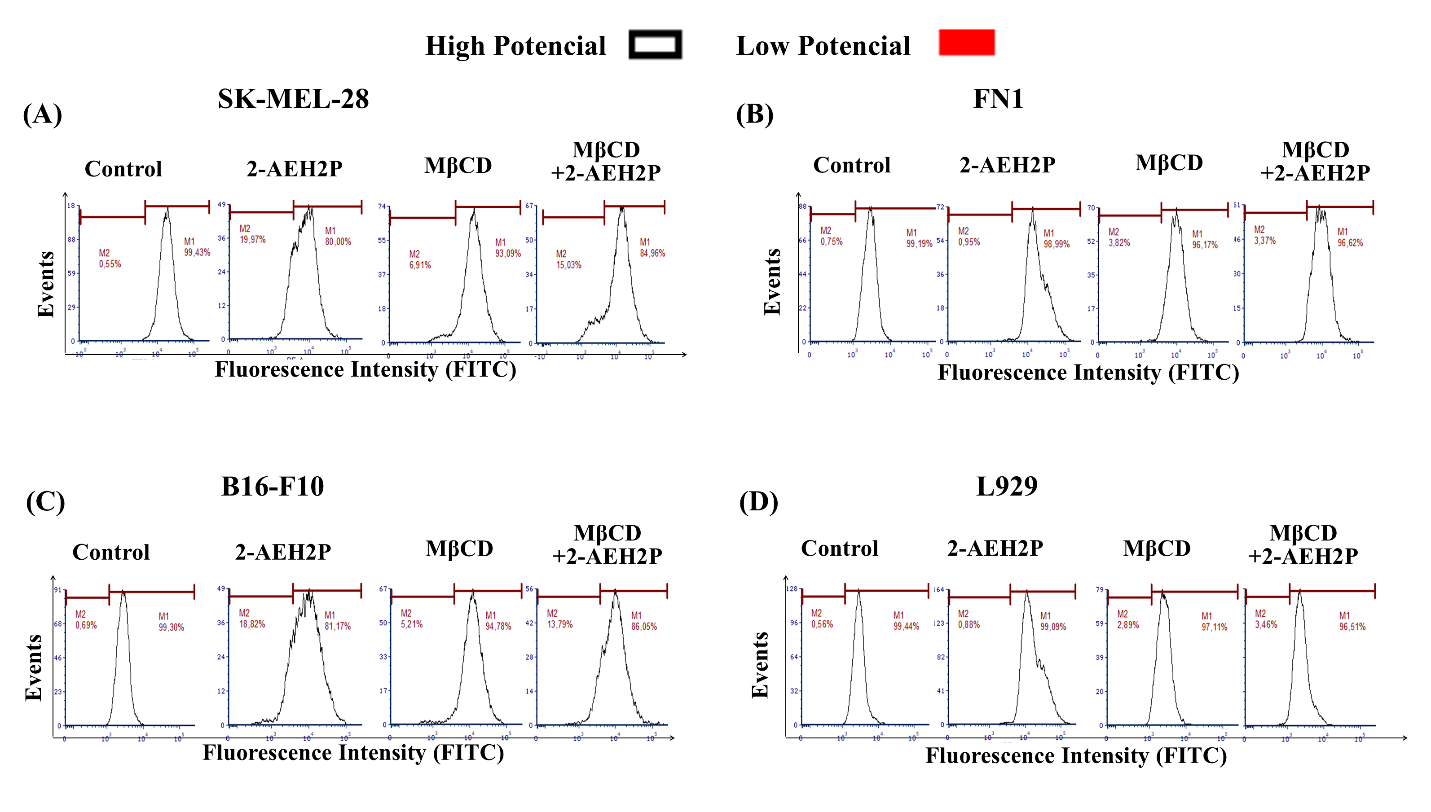


**Supplementary Figure 3.** Representative mitochondrial membrane potential profiles of melanoma cells and fibroblasts following treatment with 2-AEH₂P, MβCD, and their combination. Representative FITC-based fluorescence histograms showing mitochondrial membrane potential status in (A) SK-MEL-28, (B) FN1, (C) B16-F10, and (D) L929 cells after 24 h of treatment. High-potential populations are indicated by the open (white) gate, whereas low-potential depolarized populations are shown in red. Treatment with 2-AEH₂P, MβCD, and especially their combined regimen resulted in a marked increase in low-potential fractions in melanoma cells, consistent with mitochondrial disruption, while fibroblasts displayed comparatively modest changes. Percentages represent the proportion of cells within each potential state for each treatment condition.


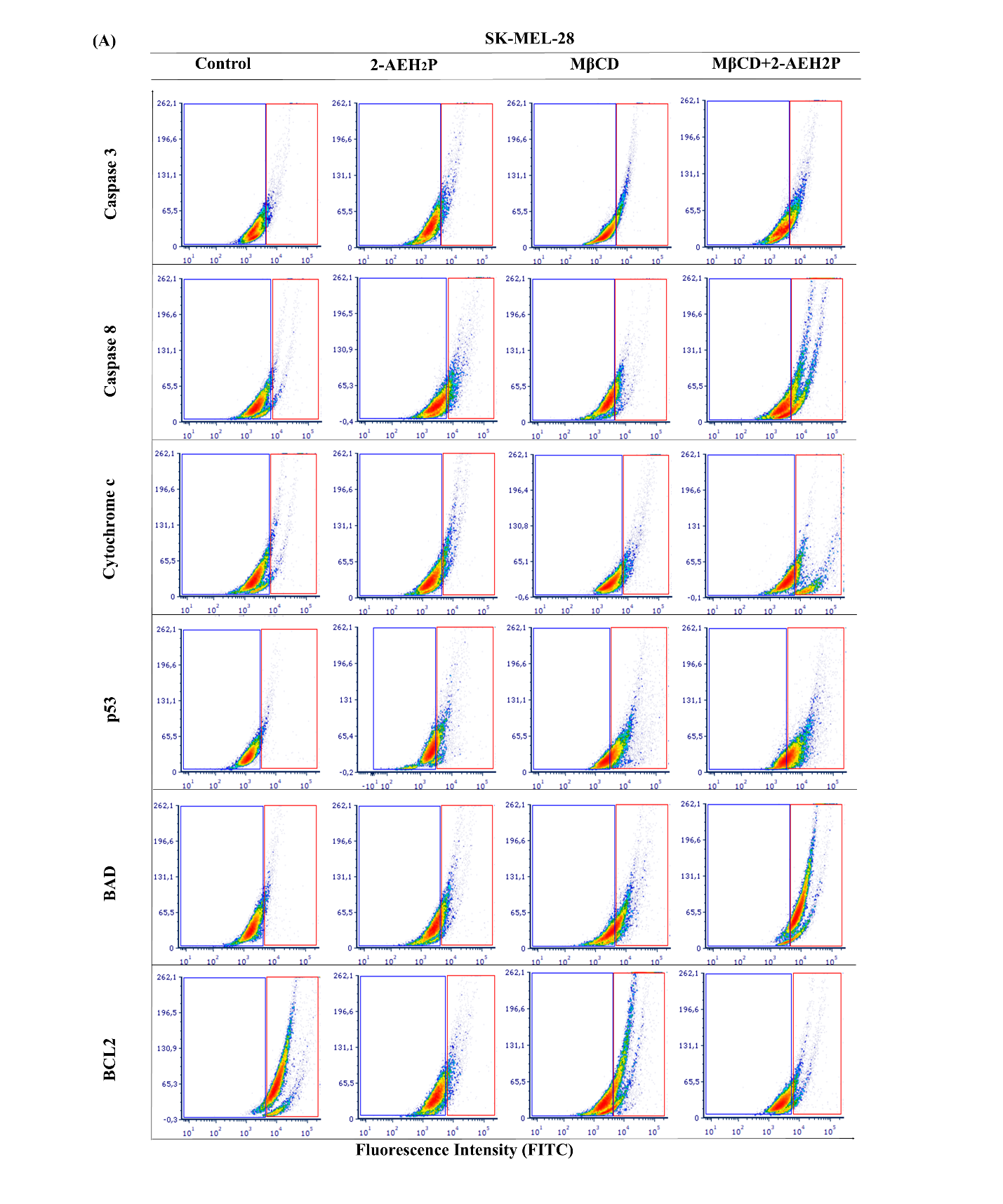


**Supplementary Figure 4.** Representative flow cytometry dot plots of apoptotic and mitochondrial markers in SK-MEL-28 cells after treatment with 2-AEH₂P, MβCD, and their combination. Representative FITC-based fluorescence plots showing the expression of activated caspase-3, caspase-8, cytochrome c, p53, BAD, and BCL-2 in SK-MEL-28 melanoma cells following 24 h of exposure to 2-AEH₂P, MβCD, or the combined regimen. Gated regions (red boxes) indicate the FITC-positive population for each marker. Increased fluorescence intensity in combination-treated cells reflects enhanced apoptotic signaling and mitochondrial dysfunction, whereas single-agent treatments show intermediate or modest shifts. Data illustrate the mechanistic contribution of both agents to apoptosis induction in melanoma cells.


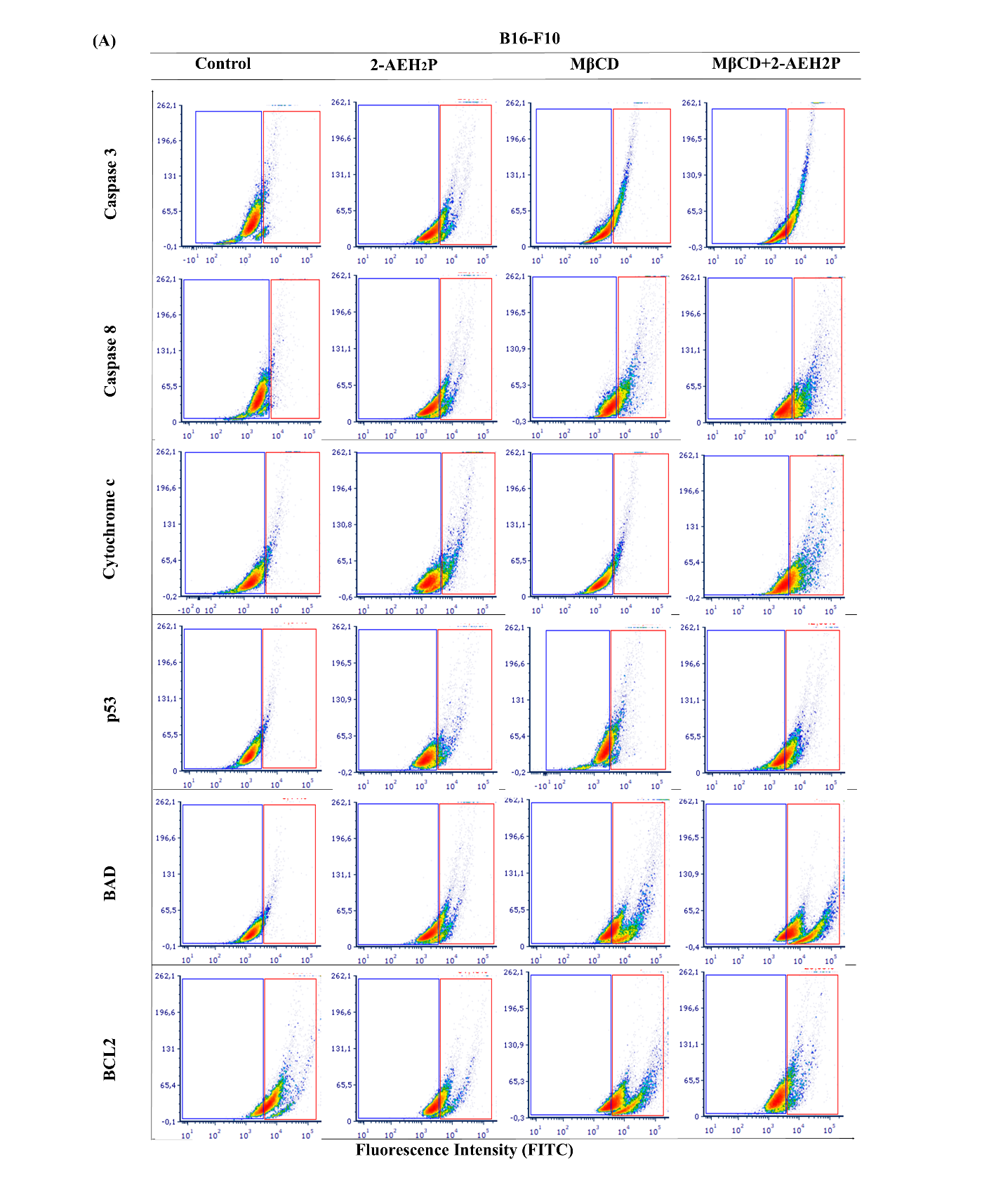


**Supplementary Figure 5.** Representative flow cytometry dot plots of apoptotic and mitochondrial markers in B16-F10 cells after treatment with 2-AEH₂P, MβCD, and their combination. Representative FITC-based fluorescence plots showing the expression of activated caspase-3, caspase-8, cytochrome c, p53, BAD, and BCL-2 in B16-F10 melanoma cells following 24 h of exposure to 2-AEH₂P, MβCD, or the combined regimen. Gated regions (red boxes) indicate the FITC-positive population for each marker. Increased fluorescence intensity in combination-treated cells reflects enhanced apoptotic signaling and mitochondrial dysfunction, whereas single-agent treatments show intermediate or modest shifts. Data illustrate the mechanistic contribution of both agents to apoptosis induction in melanoma cells.
